# Empathic Accuracy and Observed Demand Behavior in Couples

**DOI:** 10.3389/fpsyg.2016.01370

**Published:** 2016-09-12

**Authors:** Céline Hinnekens, Gaëlle Vanhee, Maarten De Schryver, William Ickes, Lesley L. Verhofstadt

**Affiliations:** ^1^Family Lab, Department of Experimental-Clinical and Health Psychology, Ghent UniversityGhent, Belgium; ^2^Learning and Implicit Processes Lab, Department of Experimental-Clinical and Health Psychology, Ghent UniversityGhent, Belgium; ^3^Department of Psychology, The University of Texas at ArlingtonArlington, TX, USA

**Keywords:** intimate relationships, empathic accuracy, demand behavior, relationship quality, conflict (psychology)

## Introduction

Disagreements and conflicts are inevitable in intimate relationships because each partner has his or her own expectations, goals, values and perspectives (Lewin, 1948). How partners behave, think, and feel during relationship conflict has been an important topic of psychological investigation for many decades (see Bradbury and Karney, 2014 for an overview). The current data-report extends existing research on this matter by providing data from a large-scale observational study on partners' empathic accuracy and demand behavior during conflict interactions.

Empathic accuracy in couples is defined as the extent to which partners understand each other's unspoken thoughts or feelings as they spontaneously occur during the course of their everyday interactions (Ickes, 2003, p. 588). Although empathic accuracy refers to one's understanding of the inner world of the other, which is a difficult process to operationalize, (Ickes et al., 1990) succeeded in developing a paradigm (i.e., the unstructured dyadic interaction paradigm) to measure the interaction partners' levels of empathic accuracy in an objective but naturalistic manner. Within this paradigm, the perceiver's level of empathic accuracy is determined by coding the degree of similarity between the target's reported thoughts/feelings during an interaction and the perceiver's inferences about each of the target's thoughts/feelings.

This paradigm has been frequently used in studies on the role of motivation in partners' level of empathic (in)accuracy. The results of these studies have provided considerable evidence that different forms of motivation—either stable long-term motives or more transient, situational-specific motives—play an important role in the perceiver's level of empathic accuracy (Ickes, 2011). This motivation may be relationship-promoting, in that a certain level of accurate insight into each other is needed when partners want to effectively coordinate their individual and shared actions (e.g., in providing adequate support, Verhofstadt et al., 2011; in reinforcing perceived closeness, Simpson et al., 2003; in accommodating behavior during relationship conflict, Kilpatrick et al., 2002; Bates and Samp, 2011). Motivation that stimulates the intention to be accurate may also stem from individual characteristics, such as being encouraged to comply with gender-role stereotypes (i.e., according to which women are more empathic; Ickes et al., 2000), or partners experiencing a sense of distrust reflected in an anxious attachment style (Dugosh, 2001).

On the other hand, there is also evidence that partners can be motivated to be *less* accurate when doing so helps to protect their relationship (Ickes and Simpson, 1997, 2001). Specifically, individuals are motivated to be less accurate when the partner is likely to be harboring thoughts and feelings which—if accurately inferred—would have a distressing and destabilizing effect on their relationship (e.g., Simpson et al., 1995, 2003). As Smith et al. (2014) have noted, intimate partners are capable of “managing” their empathic accuracy, dialing it up or down depending on the demands of the situation or their own motives.

Although relationship conflict can be perceived as a threatening or stressing event, it can also be viewed as an opportunity to reconcile partners' different goals or opinions, to expose personal needs or desires, or to express concern about the partners' inappropriate behavior or the current status of the relationship. By raising a certain topic of disagreement, partners generally want to change the status quo of the relationship or to induce a certain change in their partner's opinion or behavior (Eldridge and Christensen, 2002). In the literature, this partner is referred to as the conflict initiator or the agent of change (e.g., Christensen and Pasch, 1993). This conflict initiating partner often relies on *demanding communication*, which is defined as the tendency to demand change in a critical and blaming manner, for example, by nagging, complaining, criticizing or “pressing” the other. The other partner may react by withdrawing, reflected in avoiding the other partner or by terminating or escaping from the conflict (Christensen, 1988).

A certain level of demand-withdraw behavior is commonly observed during conflict interactions, even in satisfied couples (Eldridge et al., 2007; Baucom et al., 2010). However, a polarized pattern of demand-withdraw behavior can be associated with relationship distress, power differences—and even violence—within the relationship (Sagrestano et al., 1999), as well as with relationship dissatisfaction in the long-term (Eldridge and Christensen, 2002). Although some studies have reported a tendency for women to take the demanding role and men the withdrawing role (Christensen, 1988; Eldridge and Christensen, 2002), other studies suggest that the role of initiating a disagreement or the conflict topic *per se* is more predictive of being in the demander role than is gender (Christensen and Heavey, 1990; Heavey et al., 1993; Eldridge et al., 2007).

Consistent with the empirical evidence described above, we expect that a partner who desires change on a particular topic is likely to initiate a discussion and to behave in a way that allows him/her to bring about this desired change (i.e., by demanding). This same individual might also be motivated to accurately infer the partner's current thoughts and feelings about issue(s) at the heart of the conflict. Why? Because accurate insight into the thoughts and feelings of the partner during conflict might enable one to know what kinds of reactions to anticipate and which “buttons to push” in order to convince or change the partner.

Our study (Hinnekens et al., 2016) was the first to examine the question of whether intimate partners who are highly motivated to induce change in their partner during conflicts will be more empathically accurate than partners who are less motivated to do so. The results of this study suggested that some forms of demand behavior are indeed associated with the level of empathic accuracy during a conflict interaction, thereby confirming the assumption that perceivers who are motivated to induce changes in their partner or the relationship are also motivated to accurately infer their partner's minds in ways that enable them to exert more influence on their partner and eventually ‘push’ the partner toward the desired outcome.

The current dataset includes empathic accuracy and demand behavior data from the 310 partners of 155 couples who were observed during conflict interactions. It contributes to existing research by providing data from (a) a large sample of couples, (b) in a committed long-term relationship, and (c) it provides measures of their empathic accuracy for their partner's thoughts and feelings separately. It therefore enables researchers to further explore the associations between empathic accuracy and observed demand behavior, as well as potential moderators of this association (e.g., gender, age, relationship duration, relationship satisfaction).

## Materials and methods

### Ethics statement

The study was approved by the ethical committee of the Faculty of Psychology and Educational Sciences of Ghent University, Belgium.

### Participants

The sample consisted of the 310 members of 155 cohabiting/married heterosexual couples. The sample was recruited in the context of the “UGhent Family Lab Couple Study,” a large observational study over a period of 1 year, between 2014 and 2015. The recruitment strategy enlisted couples to volunteer for the study through posters and social media notices on the one hand and by masters'-level students in clinical psychology recruiting couples in their own vicinity on the other hand.

Couples who expressed an interest in participating were contacted by the first author, informed in general terms about the project, and evaluated to determine whether they met the inclusion criteria (i.e., being involved in their current intimate relationship for at least 1 year and being married/(partially) cohabiting for at least 6 months). Inadequate knowledge of the Dutch language and being members of same-sex couples were used as exclusion criteria. Each couple received a monetary compensation of 20€ for completing the questionnaire session and an additional 20€ for participating in the observational part of the study. Participants could withdraw from the investigation at any time and without giving any reason for their withdrawal.

The first set of measures on the online questionnaire were demographic items. The responses to these items revealed that the average reported relationship length was 12.15 years (*SD* = 11.76). The respondents' average age was 36.30 years for the men (*SD* = 14.05) and 34.21 years for the women (*SD* = 13.60), with a range of 19–76 years.

### Procedure

After providing written informed consent, the partners in each couple independently completed an internet survey. Each partner was asked to fill out this questionnaire at home in advance of the second appointment and this at their own pace, as the questionnaire could be interrupted and resumed. The questions addressed both individual—e.g., attachment style (ECR-S; Wei et al., 2007), gender identity (BSRI; Bem, 1981), general wellbeing (SHS; Lyubomirsky and Lepper, 1999; SVS; Bostic et al., 2000)—and relationship functioning—e.g., dyadic adjustment (DAS, Spanier, 1976), communication patterns (CPQ; Christensen, 1988), dyadic coping (DCI, Bodenmann, 2008) (more detailed information is available by e-mail request). The questionnaires that are relevant to the current dataset are discussed in greater detail below. Couples who completed the questionnaires were then scheduled to attend a laboratory session in which they participated in an 11-min videotaped conflict interaction task that was followed by a post-interaction video review task. At the end of their participation, the couples were fully debriefed.

## Materials

### Quality of marriage index

Relationship satisfaction was assessed with the Quality of Marriage Index (QMI, Norton, 1983). This questionnaire consists of 6 items assessing global relationship satisfaction (e.g., “My relationship with my partner is very stable”). The first five items are rated on a 7-point Likert scale (1 = *very strong disagreement* to 7 = *very strong agreement*) and the last item is rated on a 10-point Likert scale (1 = *very unhappy* to 10 = *perfectly happy*). The total score, which could range from 6 to 45 with higher scores indicating higher levels of satisfaction, was obtained by summing the scores of all the individual items. The internal consistency of the QMI was high in this sample (Cronbach's α = 0.94 for both men and women).

### The conflict interaction task

In the observational part of the study, the couples were invited to participate in a conflict interaction task that was similar to those used in previous studies of relationship conflict (e.g., Fletcher and Thomas, 2000; Verhofstadt et al., 2005). The couples were escorted into a laboratory that was furnished as a living room and was equipped so that the partners' interaction could be video-recorded with their prior knowledge. Both partners granted their permission for this recording by means of a written consent form.

In advance of their conflict discussion, the partners were separately asked to select a problem or issue from a list of common conflict topics in intimate relationships of which the source was either the partner or the relationship and which caused relationship distress or recurring disagreement. The topics (e.g., trust, intimacy, finances) were derived from previous work on sources of conflict within intimate relationships (Kurdek, 1994).

After this problem selection had occurred, the partners were randomly assigned to one of two conditions: *initiator* or *not initiator*. Operationally, this variable meant that the conflict issue which the designated initiator had selected was the one that the partners would discuss during their upcoming video-recorded interaction^1^. The initiator in each dyad was instructed to introduce the issue to the other partner so that they could discuss this problem together for 11 min. Both partners were instructed to act as much as possible as they would at home when discussing a similar problem with each other.

### Video review task

Immediately after the 11-min conflict interaction, both partners completed a video review task similar to that used in previous studies of empathic accuracy (e.g., Verhofstadt et al., 2005, 2016). The partners were seated in separate locations and asked to re-experience their interaction while they each viewed a video of their interaction on a laptop computer. The video presentation was controlled by an interactive software package specifically developed for the current study in order to facilitate the data collection (Hinnekens and Kimpe, 2014; more information is available by e-mail request).

Every 90 s, the video was paused and the same set of instructions appeared on the screen. First, each partner was asked to type the specific thought and feeling that s/he had at that point in the interaction in a blank box that appeared in the context of an online questionnaire (this questionnaire included additional multiple choice items that are not relevant to the current data set, however these items may have influenced the open-end questions)^2^. Second, each member of the couple was asked to infer the specific content of each of their partner's thoughts and feelings, and to type each of these inferences in the blank boxes that appeared on the online questionnaire (followed by parallel multiple choice items)^3^.

The instructions for all of these questions emphasized that the answer should be based on the 10-second interaction interval that immediately preceded the tape stop. To help ensure that both partners based their answers on the same 10-second time interval, our custom software program gave the participants the option to re-observe the 10-second interval that occurred right before the tape stop.

### Empathic accuracy coding

There was a pool of eleven trained, independent judges, and each subsample of the dataset was rated by four of them. They rated the degree of similarity between the content of each actual thought or feeling that one partner recorded and the content of the corresponding inferred thought or feeling that the other partner recorded. Following the recommendations of Ickes et al. (1990), the degree of similarity was in each case rated using a 3-point scale on which 0 = *different content from the actual thought or feeling*; 1 = *similar, but not the same, content as the actual thought or feeling* and 2 = *essentially the same content as the actual thought or feeling*. Overall empathic accuracy scores were then computed as a simple percentage measure of the number of “accuracy points” earned divided by the total number of “accuracy points” possible and multiplied by 100^4^. These scores were computed separately for the set of inferred thoughts and for the set of inferred feelings so that each partner received an empathic accuracy score for thoughts and for feelings separately.

The average empathic accuracy scores for the inferred thoughts and the inferred feelings and the intraclass corrrelations are shown in Table 1.

**Table 1:** **Descriptive statistics of the study variables**.

	**Men**				**Women**			
	***M***	***SD***	**Range**	**ICC**	***M***	***SD***	**Range**	**ICC**
QMI	39.56	5.36	10–45		40.11	4.96	14–45	
EA thoughts	20.33%	11.70	0–55	0.67	19.27%	11.66	0–48	0.67
EA feelings	21.29%	12.15	0–68	0.70	21.56%	12.23	0–52	0.74
Blame	2.17	1.42	1–8.67	0.75	2.52	1.73	1–7.67	0.77
Pressure for change	3.15	1.65	1–8.67	0.71	4.04	2.08	1–9	0.77

### Conflict interaction rating system

The behaviors observed in this study were rated and analyzed using the Couples Interaction Rating System (CIRS; Heavey et al., 1998). There was a pool of six trained coders, and each subsample of the dataset was rated by three of them. They rated the observed behaviors on the following two dimensions of demand behavior: (1) blame (i.e., accusations, criticism and assignment of the partner as the causal agent for the problem), and (2) pressure for change (i.e., positive/negative and implicit/explicit pressure for change in the partner). Both dimensions were rated on a 9-point Likert scale. High interrater reliabilities were achieved for the coders' ratings of both scale dimensions (see Table 1). Because of the high levels of interrater reliability, the behavioral ratings were averaged across the three raters.

### Dataset description

The data discussed in this manuscript have been deposited in FigShare and is accessible through the following hyperlink https://figshare.com/s/1dd9ca870d12284ddfb6 under the name “Empathic Accuracy and Observed Demand Behavior in Couples.” The deposit contains four files: (1) a.sav file, a.csv file and a.txt file containing all the raw and processed data (general information, item and total scores of the relationship satisfaction questionnaire, and raw and total scores resulting from the coding of the CIRS and empathic accuracy); and (2) a.docx file containing some additional information about the variables in the data file.

## Author contributions

All authors listed, have made substantial, direct and intellectual contribution to the work, and approved it for publication.

## Funding

The first author is an aspirant of the Research Foundation Flanders (FWO; grant number: 11Q9516N).

### Conflict of interest statement

The authors declare that the research was conducted in the absence of any commercial or financial relationships that could be construed as a potential conflict of interest.

## Footnotes

^1^The topic selected by the partner who was randomly designated as not initiator was not discussed during the couple's videotaped conflict interaction.

^2^The following additional multiple choice items were asked during the first part of the video review task: to (1) rate how obviously they believed their expression of the reported thoughts and feelings was in their behavior at the time; and (2) rate how threatening they perceived the content of their thoughts and feelings to be to themselves, to their partner, and to their relationship.

^3^The following parallel multiple choice items were asked during the second part of the video review task: to (1) rate how obviously they believed their partner expressed each thought or feeling in his or her behavior at the time; and (2) rate how threatening each of their partner's inferred thoughts and feelings were toward themselves, for their partner, and for their relationship.

^4^The theoretical range of this percent-correct accuracy measure was 0 (none of the possible accuracy points was earned) to 100 (all of the possible accuracy points were earned).
